# Virtual reality-based action observation facilitates the acquisition of body-powered prosthetic control skills

**DOI:** 10.1186/s12984-020-00743-w

**Published:** 2020-08-20

**Authors:** Manabu Yoshimura, Hiroshi Kurumadani, Junya Hirata, Hiroshi Osaka, Katsutoshi Senoo, Shota Date, Akio Ueda, Yosuke Ishii, Seiji Kinoshita, Kozo Hanayama, Toru Sunagawa

**Affiliations:** 1 Graduate School of Biomedical and Health Sciences, Hiroshima University, Hiroshima, Japan, 1-2-3 Kasumi, Minami-Ku, Hiroshima, 734-8551 Japan; 2 Kawasaki University of Medical Welfare, Okayama, Japan, 288 Matsushima, Kurashiki, Okayama, 701-0192 Japan; 3 Kawasaki Medical School Hospital, Okayama, Japan, 577 Matsushima, Kurashiki, Okayama, 701-0192 Japan; 4 Kawasaki Medical School, Department of Rehabilitation Medicine, Okayama, Japan, 577 Matsushima, Kurashiki, Okayama, 701-0192 Japan

**Keywords:** Virtual reality, Prosthetic control, Action observation, Motor imagery, Motor learning, Prosthesis, Amputation, Upper limb

## Abstract

**Background:**

Regular body-powered (BP) prosthesis training facilitates the acquisition of skills through repeated practice but requires adequate time and motivation. Therefore, auxiliary tools such as indirect training may improve the training experience and speed of skill acquisition. In this study, we examined the effects of action observation (AO) using virtual reality (VR) as an auxiliary tool. We used two modalities during AO: three-dimensional (3D) VR and two-dimensional (2D) computer tablet devices (Tablet). Each modality was tested from first- and third-person perspectives.

**Methods:**

We studied 40 healthy right-handed participants wearing a BP prosthesis simulator on their non-dominant hands. The participants were divided into five groups based on combinations of the different modalities and perspectives: first-person perspective on VR (VR1), third-person perspective on VR (VR3), first-person perspective on a tablet (Tablet1), third-person perspective on a tablet (Tablet3), and a control group (Control). The intervention groups observed and imitated the video image of prosthesis operation for 10 min in each of two sessions. We evaluated the level of immersion during AO using the visual analogue scale. Prosthetic control skills were evaluated using the Box and Block Test (BBT) and a bowknot task (BKT).

**Results:**

In the BBT, there were no significant differences in the amount of change in the skills between the five groups. In contrast, the relative changes in the BKT prosthetic control skills in VR1 (*p* < 0.001, d = 3.09) and VR3 (*p* < 0.001, d = 2.16) were significantly higher than those in the control group. Additionally, the immersion scores of VR1 (*p* < 0.05, d = 1.45) and VR3 (*p* < 0.05, d = 1.18) were higher than those of Tablet3. There was a significant negative correlation between the immersion scores and the relative change in the BKT scores (Spearman’s r_s_ = − 0.47, *p* < 0.01).

**Conclusions:**

Using the BKT of bilateral manual dexterity, VR-based AO significantly improved short-term prosthetic control acquisition. Additionally, it appeared that the higher the immersion score was, the shorter the execution time of the BKT task. Our findings suggest that VR-based AO training may be effective in acquiring bilateral BP prosthetic control, which requires more 3D-based operation.

## Background

Prostheses can support amputees’ activities of daily living, such as eating, dressing, and hygiene tasks. These necessary functions in daily life are strongly desired by amputees regardless of the amputation level and prosthesis type [1]. Body-powered (BP) prosthesis have higher working levels and greater durability than myoelectric prostheses and cosmetic prostheses. Therefore, they are valuable devices for users who place importance on functionality [2]. However, the overall rejection rates of BP prostheses range from 16 to 66% [3], and the risk of prosthesis abandonment is higher in patients who have not received proper training [4].

Regular BP prosthesis training facilitates the acquisition of skills through repeated practice. However, repeated training requires both time and motivation. Therefore, it is necessary to develop training methods by which prosthetic control can be acquired in a short time; skill acquisition will be easier with auxiliary tools. Huinink et al. reported that indirect grip force adjustment is effective in acquiring BP prosthetic control [5]. Another study revealed similar results for myoelectric prostheses [6]. Therefore, indirect training on BP prosthetic control may promote skill acquisition.

Action observation (AO) is an application of the phenomenon in which observing the behaviour of another person produces the same neural activity as that performed by oneself. Specific areas within the premotor, motor, and parietal cortices are activated when planning, executing, and observing cognitive motor control tasks [7]. This network of areas has been defined as the “mirror neuron system (MNS)”. Previous studies showed that AO using the MNS enhances performance, such as in upper-limb function following a stroke [8] or in patients with cerebral palsy [9], gait in patients with Parkinson’s disease [10], and function and ability following hip and knee joint surgery [11]. Observation of the smooth control of a prosthetic may thus facilitate an amputee’s acquisition of skilled BP prosthetic control.

Cusack et al. measured brain activity while prosthesis users observed the action. The conditions of the study were whether the prosthesis of the subject was matched or not matched with the model’s prosthesis [12]. The results reveal that normal planning-related activities occur during AO of a subject whose types of prosthesis are matched to the amputee’s prosthesis. In other words, it has been suggested that the type and movement of observed limbs may have an influence on the adaptation of the behaviour in the prosthesis user. These results suggest that prosthetic training after upper-limb amputation may improve prosthetic control by using an actual or simulated prosthesis similar to that of the observer.

Additionally, virtual reality (VR) simulates an environment where users’ experiences are comparable to those in the real world [13]. Previous studies have shown that VR training promotes recovery of upper-limb functions in patients with stroke or Parkinson’s disease [14–16]. Marcos et al. reported that rehabilitation using VR is a useful technology that creates the illusion of being in a virtual space and promotes better movement and cognition [17]. Therefore, indirect skill training using AO by VR may expedite the process of acquiring BP prosthetic control in upper-limb amputees.

In this study, we examined the effects of AO using VR as an auxiliary tool. We examined two different modalities during AO (VR and a tablet device (Tablet)) and two perspectives (first- and third-person views). We hypothesized that AO by VR is effective in accelerating the acquisition of BP prosthetic control. The focus of this study was whether VR is superior to tablets and whether the first-person perspective is more effective than the third-person perspective.

## Methods

### Participants

Forty right-handed participants (20 men and 20 women, 21–40 years old) volunteered for the study. None of the participants had a history of neurological or orthopaedic disorders, and all had normal or corrected-to-normal vision. They were classified as consistent right-handers according to the Edinburgh Handedness Inventory [18]; all the participants scored 90–100% on this scale. The purpose and procedures of the study were explained to the participants, and written informed consent was obtained before enrolment. The Ethical Review Board of Kawasaki Medical School (no. 3497) approved this study, and all the procedures were performed in accordance with the Declaration of Helsinki.

### BP prosthetic simulator

**Fig. 1:**
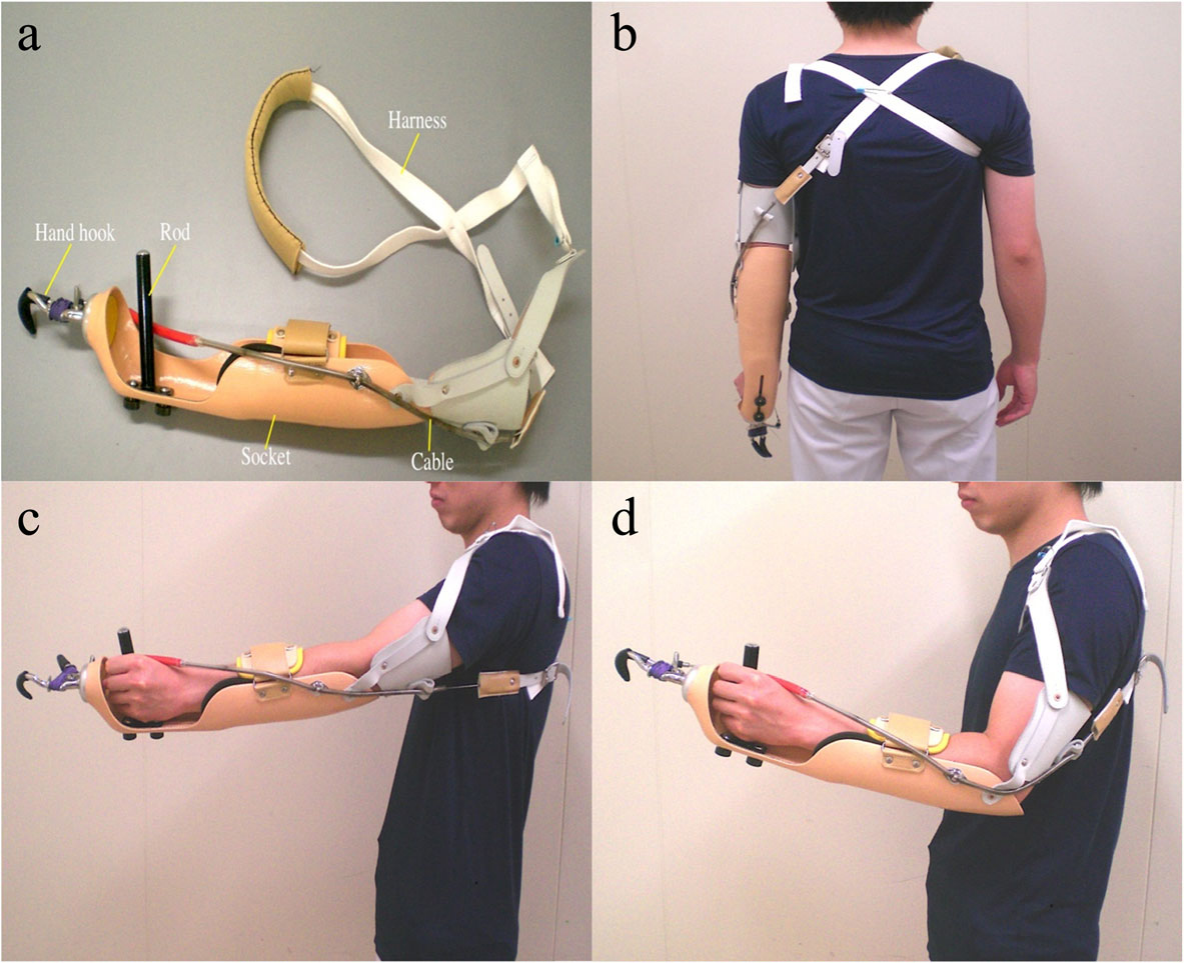
Body-powered (BP) prosthetic simulator. **a.** The BP prosthetic simulator consists of a hand hook, rod, socket, cable, and harness. **b.** The simulator was worn on the left upper limb. The simulator was controlled by a cable connected to a figure-8-shaped shoulder harness. **c, d.** The simulator is a voluntary-opening BP prosthesis. The hand hook opens with left shoulder flexion and scapular abduction and closes with left shoulder extension and scapular adduction

A BP prosthetic simulator (Fig. 1a) was used to evaluate prosthetic control. None of the participants had previous experience with BP prosthetic simulators. We used a voluntary-opening BP prosthesis consisting of a hand hook, socket, rod, cable, and harness. The simulator was controlled by a cable attached to a figure-eight shoulder harness, which was then wrapped around the contralateral shoulder (Fig. 1b). The simulator on the left upper limb, as achieving effective prosthetic control with the non-dominant hand is highly difficult and indicates new motor learning. While wearing the simulator, the participant grasped the rod with their left hand; their left forearm was attached to the socket, and the harness was attached to their right shoulder. Thus, the hand hook opened with left shoulder flexion and scapular abduction and closed with left shoulder extension and scapular adduction (Fig. 1c, d). A skilled occupational therapist adjusted the harness tension and hook direction to adapt the prosthesis to each participant.

### Evaluation of functional performance

We used two functional tasks to examine unilateral and bilateral manual dexterity during prosthesis use: the Box and Block Test (BBT) [19] and a bowknot task (BKT). For the BBT, participants stood in front of a box divided into two square compartments on a stable table with one of the compartments containing 150 wooden blocks (2.5 cm^2^). The height of the table was set to the height of the participant’s navel. A non-slip mat was placed under the BBT apparatus. The participants were instructed to transport as many blocks as possible from one compartment to another with only their left-hand hook; the test score was the number of blocks transferred within 60 s [20].

**Fig. 2:**
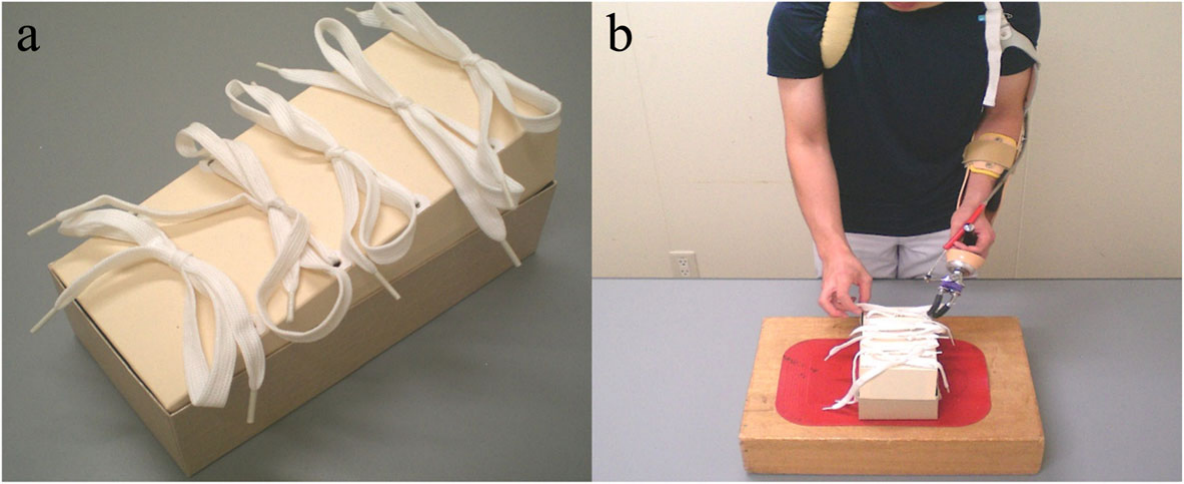
Bowknot task (BKT). **a.** The BKT was used in the evaluation of bilateral manual dexterity during prosthetic use. The BKT kit consisted of a box (20 cm × 10 cm × 15 cm) with five 15-cm-long shoelaces on each side. **b.** The participants stood in front of the box placed on a stable table and knotted the five shoelaces as fast as possible from far to near with the left-hand hook and normal right hand. The BKT score was the time need to the five shoelaces

We used the BKT to evaluate bilateral manual dexterity during prosthesis use. (Fig. 2). The BKT kit consists of a box (20 cm × 10 cm × 15 cm) and five 15-cm-long shoelaces placed on a table. The height of the table was again set to the height of the participant’s navel and a non-slip mat placed under the box. Participants stood in front of the box and knotted the five shoelaces as fast as possible from the far to the near side of the box using the left-hand hook and normal right hand. The BKT score was the time needed to knot the five shoelaces.

The BBT and BKT scores were measured twice in each session, and the mean values of each test were used as the final scores. Participants practised for five minutes before the start of each of the two tasks.

### Immersion evaluation

The visual analogue scale (VAS) was used to evaluate the immersion level during AO using a VR system and tablets. After AO, the participants provided an immersion score for the immersive visual experience elicited by the AO. We obtained the immersion score using the VAS, which ranged from 0 (not completely immersive) to 100 (completely immersive). The final immersion score was the average of the immersion scores during the two video observations.

### Video image and viewing

The participants observed a video of a real person wearing a BP prosthetic simulator and performing the BBT and BKT smoothly. The model person in the video was well practised and had good operating skills. Based on the participants’ pre-intervention (Pre) data, the BBT film demonstrated carrying 30 blocks per minute, and the BKT file demonstrated knotting five shoelaces in 80 s. Each film ran repeatedly for 10 min during each session.

We recorded four videos for AO training: two 3D videos for viewing in the VR system and two 2D videos for viewing on the tablet. The two VR videos were recorded using a VR camera (Mirage Camera with Daydream, Lenovo, China). The two tablet videos were recorded using the tablet’s camera (Huawei dtab d1-01H, Huawei, China).

**Fig. 3:**
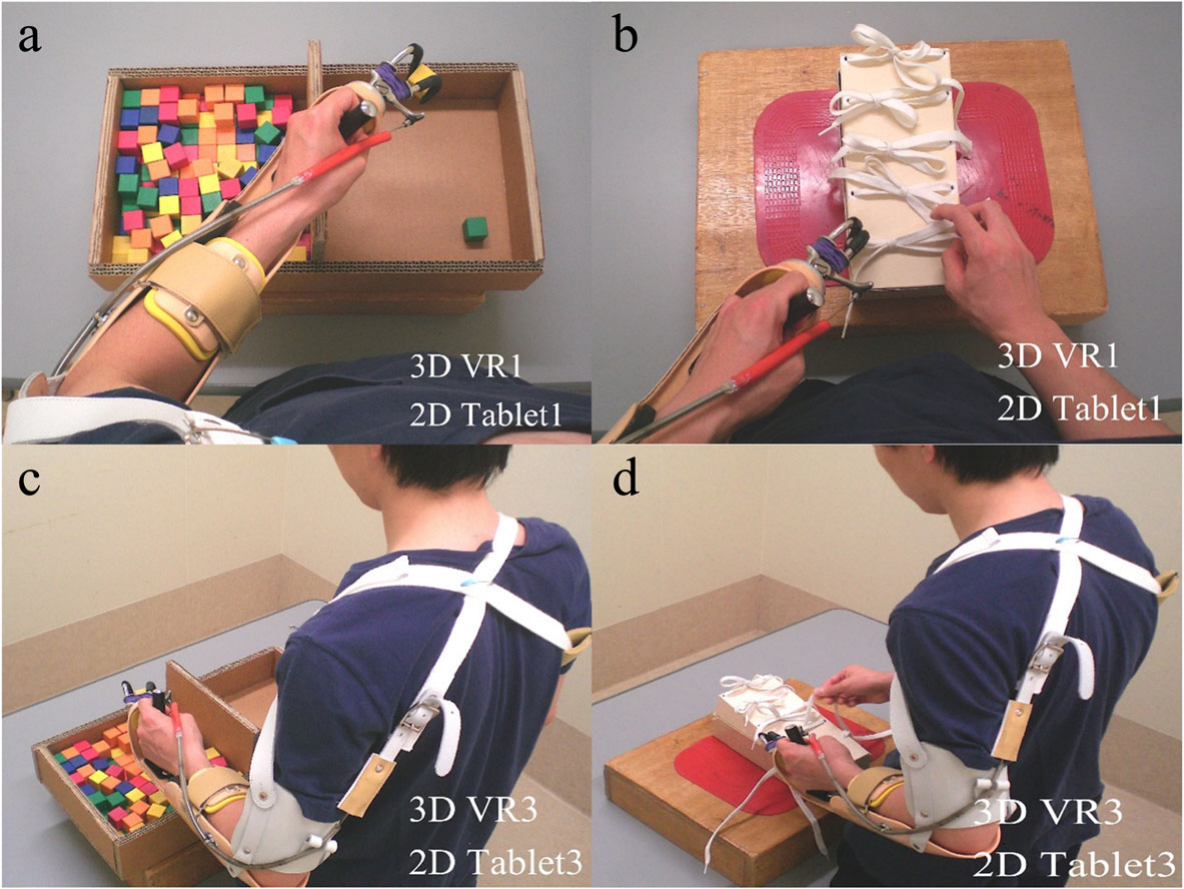
VR1, VR3, Tablet1, and Tablet3 group video images. **a, b**. The VR1 and Tablet1 videos were a point of view as if one were performing the prosthetic operation (first-person perspective). The VR1 group watched a 3D video; the Tablet1 group watched a 2D video. **c, d**. The VR3 and Tablet3 videos showed a real person performing a prosthetic operation where the movement of the scapula to the forearm was easy to observe (third-person perspective). The VR3 group watched a 3D video; the Tablet3 group watched a 2D video

The first (3D) VR video showed a first-person perspective of a real person performing the BBT and BKT (Fig. 3 a, b); the participant’s perspective and arm position were adjusted to match the video. During observation, it was confirmed that none of the participants complained of discomfort or confused their hands. The second (3D) VR video showed a third-person perspective of a real person performing the tasks (Fig. 3 c, d). The first (2D) tablet video is a first-person perspective video (Fig. 3 a, b), and the second (2D) tablet video is a third-person perspective video (Fig. 3 c, d). The same person was shown in videos shot in the first- and third-person perspectives.

Participants’ viewing of the VR films used a head-mounted display (Mirage Solo with Daydream, Lenovo, China). The tablets used for viewing the first- and third-person videos were equipped with 10.1-in. displays.

### Intervention group

**Fig. 4:**
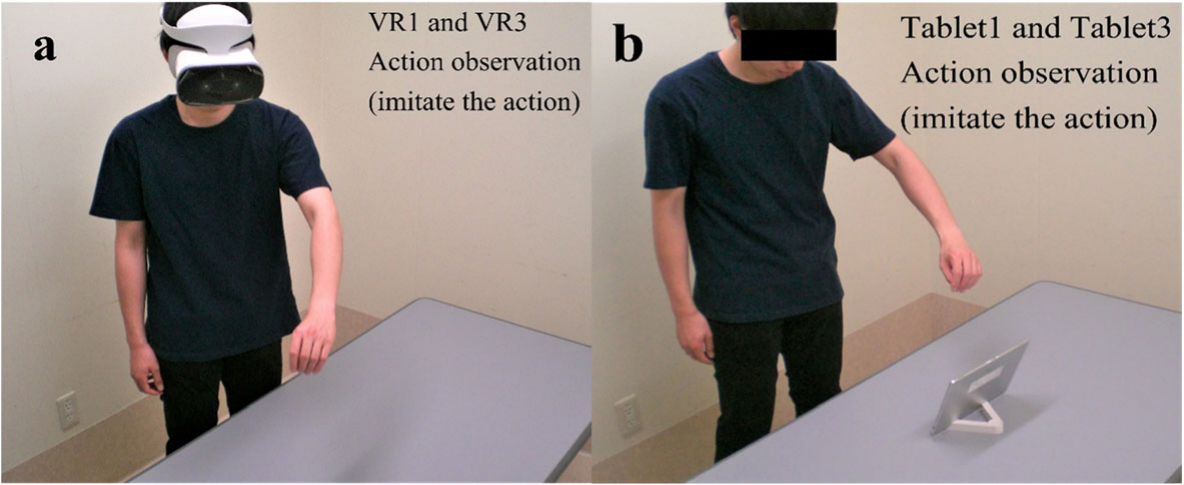
Imitation of movement during action observation (AO). **a.** AO scene for VR1 and VR3, who imitated the motion of the video while observing the 3D VR video. **b.** AO scene of Tablet1 and Tablet3, who imitated the motion of the video while observing the 2D video on a tablet

The participants were divided into four intervention groups (VR1, VR3, Tablet1, and Tablet3) and one control group, with each group consisting of eight participants. During the video observation period, the participants removed the BP prosthetic simulator and imitated the movement, imagining that they were actually operating a prosthesis (Fig. 4).

1) In the VR first-person perspective group (VR1), the VR video was viewed using a head-mounted display. The participants watched the VR video in 3D 180° vertically and horizontally. The participants watched the 3D videos of prosthesis skills and imagined they were performing the prosthetic operations. The video showed the point of view as if one were performing a prosthetic operation (first-person perspective) (Fig. 3a, b).

2) In the VR third-person perspective group (VR3), participants watched 3D videos of prosthesis skills and imagined they were performing the prosthetic operations. The video showed the viewpoint from which movement of the scapula to the forearm was easy to observe when performing the prosthetic operation (third-person perspective) (Fig. 3c, d).

3) In the first-person perspective group using a tablet (Tablet1), the participants watched the 2D videos of prosthesis skills and imagined they were performing the prosthetic operations. The video was from the point of view as if one were performing a prosthetic operation (first-person perspective) (Fig. 3a, b).

4) In the third-person perspective group using the tablet (Tablet3), the participants watched the 2D videos of prosthesis skills and imagined they were performing the prosthetic operations. The video was shot from a viewpoint from which the movement of the scapula to the forearm was easy to observe when operating the prosthesis (third-person perspective) (Fig. 3c, d).

5) The fifth group was the control group (Control). This group was evaluated on the BBT and BKT without intervention.

### Protocol (Fig. 5)

We used the BBT and BKT as the pre-intervention (Pre) and post-intervention (P1 and P2) measures. The first and second rounds of the interventions were labelled Session 1 (S1) and Session 2 (S2), respectively. The relative change in the BBT scores is the increase in the BBT scores from P1 and P2 when the Pre score is set to zero. The relative change in the BKT scores is a decrease in the BKT scores from P1 and P2 when the Pre score is set to 100. The final immersion score is the average of the immersion scores during the video observation periods of S1 and S2. The intervention groups watched the BKT and BBT videos for 10 min in each session as outlined in the study by Cusack et al. [12]. During the video observation periods, the participants removed the BP prosthetic simulator and imitated the movement as if they were operating a prosthesis (Fig. 4). We performed the BBT and BKT after the first and second interventions, which were Post 1 (P1) and Post 2 (P2), respectively. We obtained immersive evaluation scores at P1 and P2 in the four experimental groups: VR1, VR3, Tablet1, and Tablet3.

**Fig. 5:**
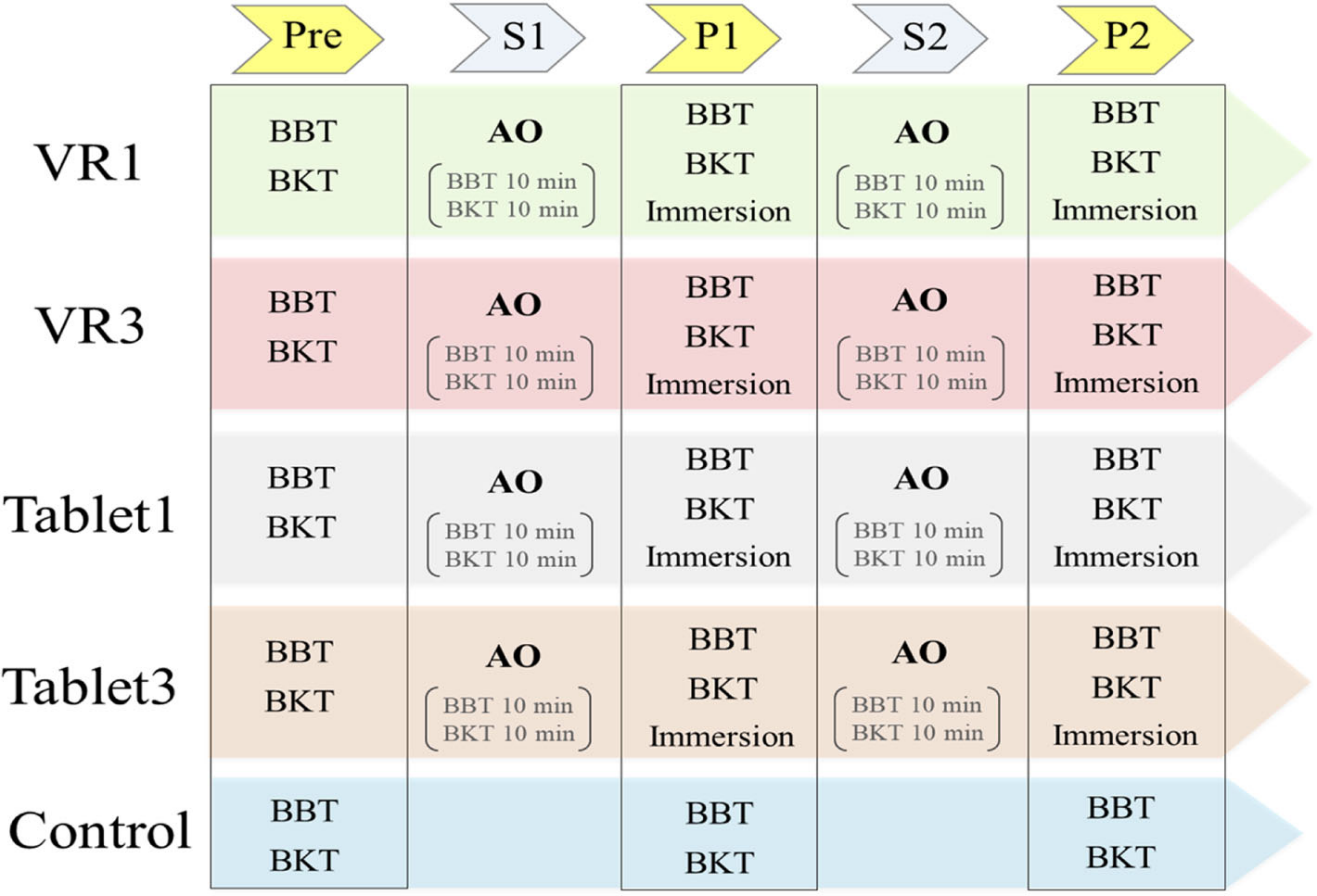
Protocol. Pre, pre-intervention; S1, Session 1; S2, Session 2; P1, Post 1; P2, Post 2; BBT, Box and Block Test; BKT, bowknot task; AO, action observation. Before the intervention (Pre), the BBT and BKT were used to evaluate prosthetic performance. Then, the participants were divided into five groups (VR1, VR3, Tablet1, Tablet3, and Control) for the intervention. After the intervention, the same evaluation as in the pre-intervention was conducted. Furthermore, with the exception of the control group, immersion during action observation and motor imagery was evaluated. This protocol was repeated twice

### Statistical analysis

To analyse the effects of intervention, we used the following: 1) the relative change the BBT scores, 2) the relative change in the BKT scores, and 3) the immersion score. Initially, the normality of the data was confirmed using the Shapiro-Wilk test. After normality was confirmed, the relative changes in the BBT and BKT scores were analysed using two-way ANOVA for groups VR1, VR3, Tablet1, Tablet3, and Control and sessions (Pre, P1, and P2). The immersion scores were analysed using one-way ANOVA of groups VR1, VR3, Tablet1, and Tablet3. Holm’s correction was used for post hoc comparisons when significant main effects were found. The effect sizes for the respective measurements were assessed using Cohen’s d; d > 0.8 indicates a large effect size. We further investigated the relationships between the relative change scores for the BBT and BKT (Pre to P2) and immersion score using Spearman’s rank correlation coefficient. All the results are reported as means ± standard deviations. Statistical analysis was performed using IBM SPSS version 23.0 (IBM japan, Tokyo, Japan). The significance level was set at 5%.

## Results

### Participants

The distributions of participants’ sex, age and Edinburgh Handedness Inventory score for each group are shown in Table 1. There were no significant differences in these factors between groups.

**Table 1:** Participants’ demographic data

	VR1	VR3	Tablet1	Tablet3	Control
Participants	8	8	8	8	8
Sex (Male/female)	4 / 4	4 / 4	4 / 4	4 / 4	4 / 4
Age Mean (SD)	25.4 (3.7)	26.1 (5.9)	27.9 (6.9)	25.1 (4.9)	27.1 (4.0)
Edinburgh Handedness Inventory score	98%	98%	93%	98%	94%

Mean (SD), *SD*  standard deviation

This table shows the demographic data of sex, age, and Edinburgh Handedness Inventory score. No significant differences in age and Edinburgh Handedness inventory score between groups were found

### Unilateral manual dexterity: the BBT

**Fig. 6:**
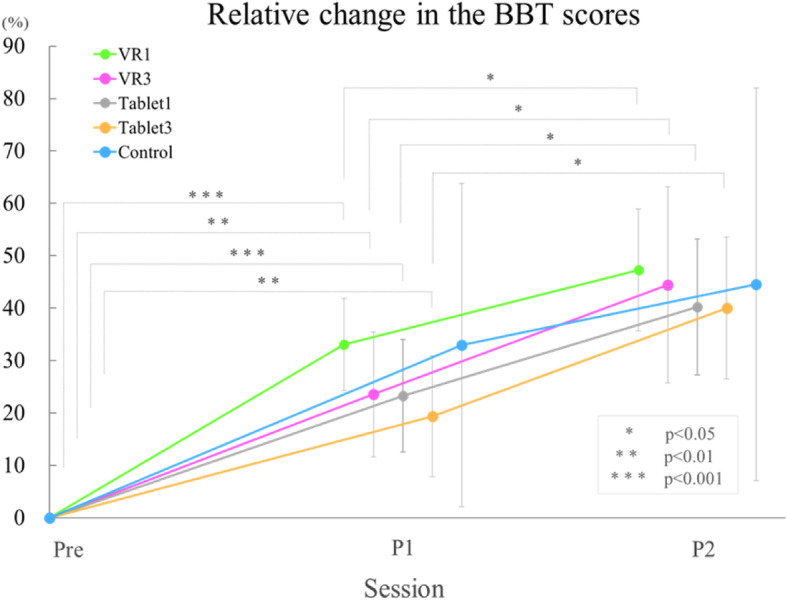
Relative change in the Box and Block Test (BBT) scores. The graph shows the relative change in the BBT scores. This graph shows the relative change in the BBT scores of P1 and P2 based on the Pre score. The means (± SDs) are shown on the graph. The vertical axis of the graph shows the relative change from Pre. A significant main effect of session was found (F(2, 14) = 109.769 *p* < 0.001); however, there was no significant main effect of group in the ratio (F(4, 28) = 0.424, *p* = 0.790), nor was the interaction significant (F(8, 56) = 0.512, *p* = 0.842)

The results of the relative change in the BBT scores are shown in Table 2. The relative change in the BBT scores is an increase in the BBT scores of P1 and P2 when Pre is set to zero. The pre-BBT score was not significantly different between the groups. The number of transported blocks increased between sessions in all five groups. The changes in the BBT relative changes are shown in Fig. 6. A significant main effect of session was found (F(2, 14) = 109.769, *p* < 0.001); however, there was no significant main effect of group in the ratio (F(4, 28) = 0.424, *p* = 0.790) nor was the interaction significant (F(8, 56) = 0.512, *p* = 0.842). Thus, all the participants experienced a significant increase in unilateral manual dexterity with increasing sessions. However, there were no differences in the acquisition level between the groups.

**Table 2:** The relative change in the Box and block test scores

Relative change in the BBT scores (%)	Pre	P1 Mean (SD)	P2 Mean (SD)
**VR1**	0	33.1 (8.8)	47.3 (11.6)
**VR3**	0	23.6 (11.9)	44.5 (18.7)
**Tablet1**	0	23.3 (10.7)	40.2 (12.9)
**Tablet3**	0	19.4 (11.5)	40.0 (13.6)
**Control**	0	33.0 (30.8)	44.6 (37.4)

Mean (SD), SD = standard deviation

This table shows the relative change in the BBT scores in P1 and P2 based on the Pre score. A significant main effect for sessions was found (F (2, 14) = 109.769 p < 0.001); however, there was no significant main effect for groups in the ratio (F (4, 28) = 0.424, p = 0.790), nor was the interaction significant (F (8, 56) = 0.512, p = 0.842)

### Bilateral manual dexterity: the BKT

**Fig. 7:**
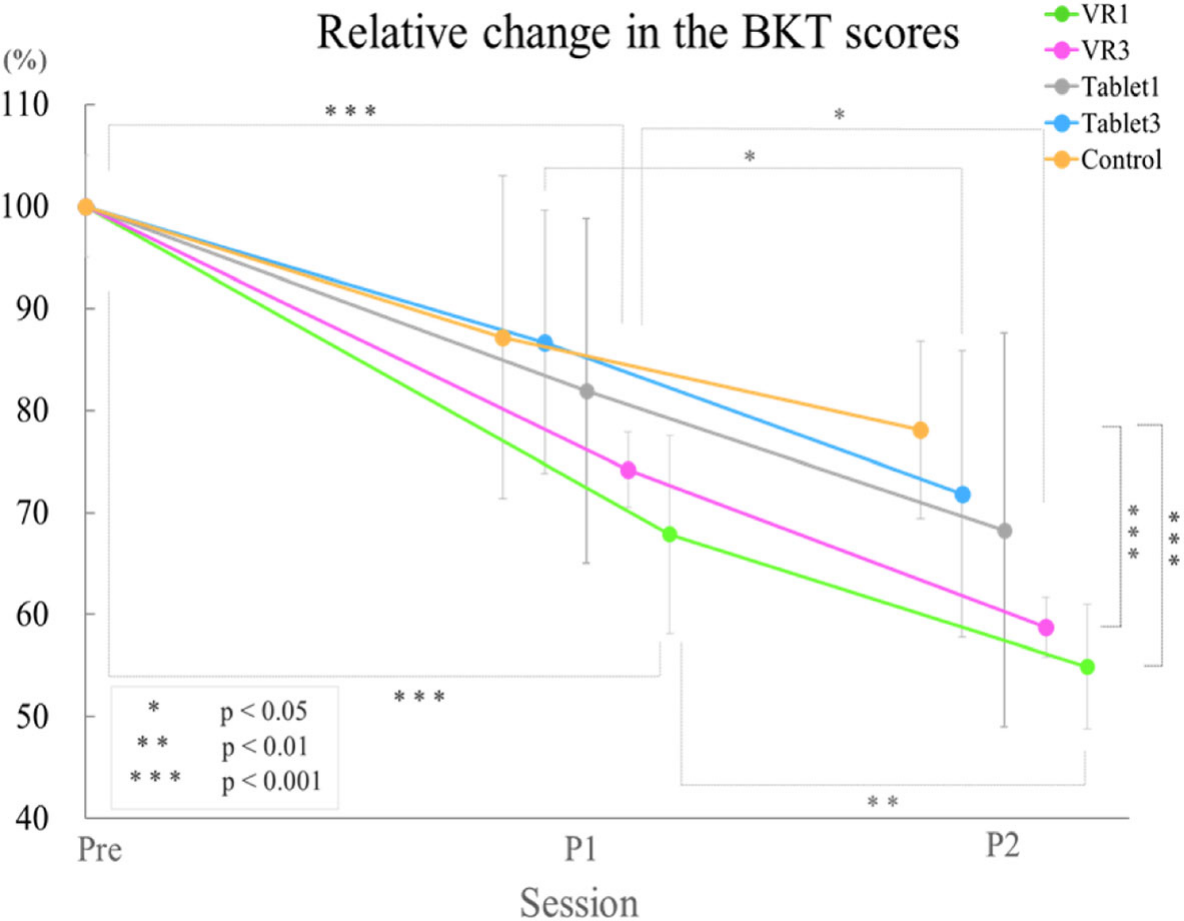
Relative change in the bowknot task (BKT) scores. The graph shows the relative change in the BKT scores. This graph shows the relative change in the BKT scores of P1 and P2 based on the Pre score. The means (±SDs) are shown on the graph. The vertical axis of the graph shows the relative change from Pre. There was a main effect of group (F(4, 28) = 3.792, *p* = 0.014). Among the groups, VR1 (*p* < 0.001, d = 3.09) and VR3 (*p* < 0.001, d = 1.18) showed higher change degrees than the control. There was a main effect of group among the sessions (F(2, 14) = 176.935 *p* < 0.001). The two-way interaction between group and session was not significant (F(8, 56) = 1.863, *p* = 0.084)

The relative changes in the BKT scores are shown in Table 3. The pre-BKT scores were not significantly different between the groups. All five groups tended to complete the test in less time with increasing sessions. The changes in the BKT relative changes are shown in Fig. 7. The two-way ANOVA indicated significant differences in the relative change in the main effect of session (F(2, 14) = 176.935, *p* < 0.001) and group (F(4, 28) = 3.792, *p* = 0.014); the two-way interaction between group and session was not significant (F(8, 56) = 1.863, *p* = 0.084). Post hoc tests showed that VR1 (*p* < 0.001, d = 3.09) and VR3 (*p* < 0.001, d = 2.16) had significantly higher rates of change compared to Post2 in the control group. Thus, VR1 and VR3 showed significantly better acquisition of bilateral prosthetic control skills compared to the control group.

**Table 3:** Relative change in the Bowknot task scores

Relative change in the BKT scores (%)	Pre	P1 Mean (SD)	P2 Mean (SD)
**VR1**	100	67.9 (9.7)	54.9 (6.1) *^1^
**VR3**	100	74.2 (14.0)	58.8 (9.2) *^2^
**Tablet1**	100	81.9 (16.9)	68.3 (19.3)
**Tablet3**	100	86.7 (12.9)	71.8 (14.0)
**Control**	100	87.2 (15.8)	78.1 (8.7) *^1^ *^2^

Mean (SD), SD = standard deviation

*^1)^ VR1 vs Control *p* < 0.001, d = 3.09 *^2)^ VR3 vs Control *p* < 0.001, d = 2.16

This table shows the relative change in the BKT scores in P1 and P2 based on the Pre score. There was main effect of group (F (4, 28) = 3.792, *p* = 0.014). Among groups, VR1 (*p* < 0.001, d = 3.09) and VR3 (*p* < 0.001, d = 2.16) showed higher rates than the Control. There was main effect of sessions (F (2, 14) = 176.935 *p* < 0.001). The two-way interaction between groups and sessions was not significant (F (8, 56) = 1.863, *p* = 0.084)

### Immersion

**Fig. 8:**
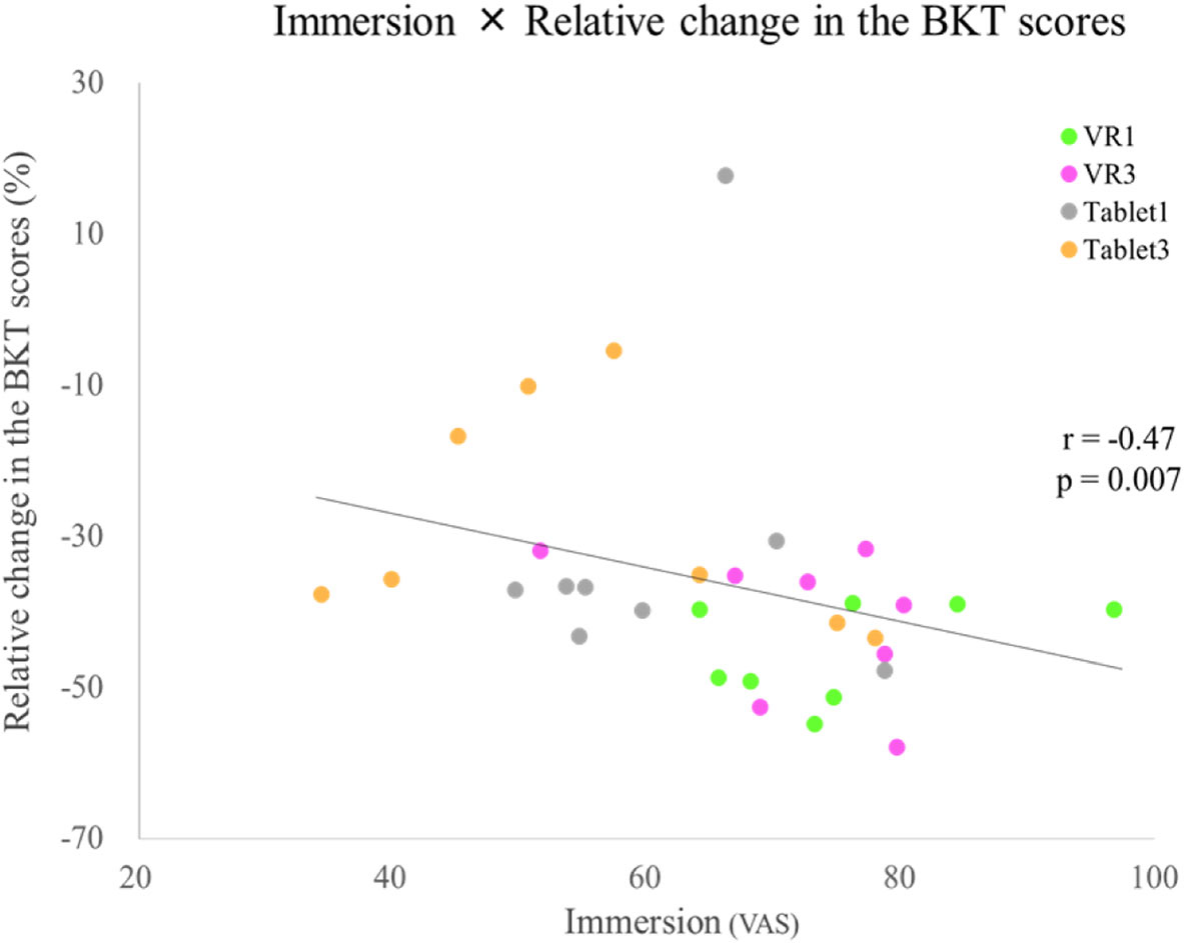
Correlation between immersion and the relative change in the bowknot task (BKT) scores. The graph shows the correlation between immersion and the relative change in the BKT scores. The vertical axis of the graph shows the relative change from Pre. The horizontal axis of the graph shows the immersion score. There was a significant negative correlation between immersion score and the relative change in the BKT scores (Spearman’s r_s_ = − 0.47, *p* = 0.007)

The immersion scores of the four groups are shown in Table 4. The one-way ANOVA indicated significant differences between groups (F(3, 28) = 4.916, *p* = 0.007). Subsequent post hoc tests revealed a significant difference between Tablet3 and VR1 (*p* = 0.012, d = 1.45) and between Tablet3 and VR3 (*p* = 0.045, d = 1.18). The results of the correlation test between immersion score and relative change in the BKT scores (Pre to P2) are shown in Fig. 8. There was a significant negative correlation between immersion score and the relative change in the BKT scores (Spearman’s r_s_ = − 0.47, *p* = 0.007). There was no correlation between immersion and the relative change in the BBT scores.

**Table 4:** Immersion scores

Immersion scores	VR1	VR3	Tablet1	Tablet3
Mean (SD)	75.2 *^1^ (10.9)	71.8 *^2^ (11.5)	60.8 (11.7)	55.4 *^1^ *^2^ (15.94)

Mean (SD), SD = standard deviation

*^1)^ VR1 vs Tablet3 *p* < 0.05, d = 1.45 *^2)^ VR3 vs Tablet3 *p* < 0.05, d = 1.18

The table shows the immersion scores of action observation (AO) in each intervention. The one-way ANOVA indicated significant differences between groups (F (3, 28) = 4.916, *p* = 0.007); Subsequently, post hoc tests between the four groups showed significant difference between Tablet3 and VR1 (*p* = 0.012, d = 1.45) and between Tablet3 and VR3 (*p* = 0.045, d = 1.18)

## Discussion

In this study, AO using VR was more effective in acquiring BP prosthetic control compared to the control group. The results depended on the task, with significant differences seen in the bilateral but not the unilateral manual dexterity tasks (the BKT and BBT, respectively). Furthermore, VR1 and VR3 had higher immersion scores than Tablet3. There was a significant negative correlation between immersion score and the relative change in the BKT scores.

### Effect of AO using VR

The relative change in the BKT scores was significantly higher in VR1 and VR3 than in the control group (Fig. 7). This result suggested that AO using VR facilitates the acquisition of bilateral prosthetic control. Additionally, the immersion score was higher in VR1 and VR3 than in Tablet3 and there was a significant negative correlation between immersion score and the relative change in the BKT scores (Fig. 8). Thus, the VR system is more immersive than the tablet system, and higher immersion scores were significantly associated with shorter BKT execution times. These results indicate that VR improves immersion during AO as well as the acquisition of prosthetic control skills. This finding supports the results of Crosbie et al., who reported that a higher degree of immersion led to better reach and search movements for virtual objects in patients with stroke [21]. Using VR can increase the patients’ motivation to participate in rehabilitation and treatment tasks [22]. Rohrback et al. reported that “enjoyment”, “motivation”, and “engagement” are involved when using VR [23]. Lewis et al. noted that the level of engagement and motivation in performing tasks is a factor in determining the success of rehabilitation interventions using VR [24]. From these studies, it is likely that the use of VR improves participants’ experiences during AO and facilitates motor learning. However, these indicators have not yet been formally evaluated.

Levin et al. stated that rich virtual environments may improve motor learning by manipulating practice conditions to explicitly involve motivational, cognitive, motor control, and sensory feedback-based mechanisms. Specifically, tasks practised within a virtual environment enhance motor skill learning by integrating multiple sensory processes, such as intrinsic receptive, visual, auditory, and vestibular information, with the engagement of cognitive processes [25]. In the VR environment of the current study, participants imitated the motion during observation, which resulted in feedback of an intrinsic receptive and vestibular sensation synchronized with the video. The visual feedback of the highly immersive 3D video may also have facilitated motor learning.

There were no significant differences between the BBT and BKT when comparing VR1 and VR3. There was, however, a correlation between the immersion score and the relative change in the BKT scores (Fig. 8). In other words, the first-person and third-person VR yielded the same immersion score level, so there were no actual differences in the participants’ sensations. However, VR1 had a higher effect size than VR3 (d = 3.09 vs. d = 2.16) and appeared to promote the acquisition of BP prosthetic control more than VR3. Jackson et al. showed that imitation during AO in the first-person view activates motor-related areas more than in the third-person view and reported better integration of kinaesthetic information [26].

The reason for imitating motion during AO in this study is based on the work of Hotz-Boendermaker et al. These researchers reported that motor imagery and motor imitation are continuums of the same phenomenon with only quantitative fluctuations [27]. In addition, observed motor imagery and imitation appear to promote activity in similar motion-related regions. It has also been reported that when motor function is intact, activation of the motion-related area can be expected more by action imitation than by simple observation [28]. Based on the results of these studies, we directed participants to imitate the motions during the AO process.

### VR effect depending on task difficulty

The relative change in the BBT scores was not significantly different among the study groups (Fig. 6). This result shows that, due to the characteristics of the BBT task, VR and tablet interventions have little effect on unilateral prosthetic control skills. The evaluation of unilateral manual dexterity during prosthesis use includes the BBT, the Nine Hole Peg Test and the Southampton Hand Assessment Procedure (SHAP) [29]. The BBT is a widely used test of unilateral dexterity that has been shown to be reliable in prosthesis evaluation [30]; it can be performed in a short amount of time, and the results are simple to interpret. The BBT score is the number of blocks moved in 1 min by the one hand. Previous studies have reported an age-averaged score for the BBT in healthy adults [19, 20]. However, no study on the BBT has shown the average age prosthetic users of BBT for. Previous studies have used an average of 25–30 blocks when assessing prosthetic control skills with the BBT [31, 32]. In this study, the average P2 score for each group was 25–28 blocks with and without intervention, which was similar to the average score in previous papers. The results suggested that the BBT was easier for the participants to perform and improved prosthetic control regardless of the method of intervention.

In contrast, the relative change in the BKT scores was significantly different among the groups (Fig. 7). The BKT is a novel evaluation method in this study and evaluates bilateral manual dexterity. In the BBT tasks, the prosthetic hook needs to open and close and be transported to the same compartment each time. In contrast, the BKT is a more difficult task requiring cooperation and smooth movements between the non-amputated hand and the opposite orthotic hook. From this result, it is presumed that AO using VR is likely to be effective in more difficult tasks and those that require the cooperation of both hands.

In daily life, prosthetic users tend to perform many one-handed operations using the non-amputated hand. However, tying shoelaces, cooking, washing, hobbies, etc., require the ability to move bilaterally and cooperatively with the prosthesis. Biddiss identified activities of high need for prosthetic users, such as household maintenance and heavy lifting; sports; daily activities, such as cooking, eating, and dressing; and hobbies, such as playing a musical instrument; most of these activities involve using both hands [1]. Therefore, it is suggested that using VR as an auxiliary tool is beneficial for prosthetic users.

The BKT is a very easy test to implement and to compare the times required before and after applying an intervention, as tying by means of a prosthesis is one of the most frequent performed activities in an amputee’s daily life. Therefore, the BKT was used in the present study as a novel ambidexterity evaluation method. At present, reference values and the reliability of the BKT are not available, and we will examine these aspects in future studies.

### Comparison of VR and tablet immersion

Immersion is usually assessed with a Likert scale. The downside of Likert scales is that the choices are ambiguous and not evenly spaced. Therefore, in this study, we used the VAS, which allows a detailed evaluation of immersion using numerical values ranging from 0 (not at all immersive) to 100 (completely immersive).

VR1 (*p* < 0.05, d = 1.45) and VR3 (*p* < 0.05, d = 1.18) had higher immersion scores during the AO phase than Tablet3 (Table 4). Additionally, there was a significant negative correlation between immersion and the relative change in the BKT scores (Fig. 8), with higher immersion scores resulting in shorter task execution times. This result is consistent with those of many previous studies [26, 33]. The VR system used in this study involved a head-mounted display, which can block outside visual stimuli. As tablets cannot completely block outside stimuli, it can be assumed that the tablets made it more difficult for the participants to immerse themselves in the videos. It has been reported that immersive features of technology can have a significant impact on experience [34]. VR videos viewed with a head-mounted display has a higher spatial presence and is more immersive than VR videos viewed using a desktop computer [35]. For these reasons, VR is more immersive than 2D movies such as those on tablets, and viewers feel as if they are in a video. Our results suggest that using VR for AO could provide a higher immersion score and promote the acquisition of BP prosthetic control.

### Clinical significance

The combination of VR and prosthetic training in upper-limb amputees can facilitate short-term prosthetic control skills. As a result, our findings predict that rejection of a prosthesis in the early stage of prosthesis training can be reduced, leading to an increase in continuous use.

However, Chadwell et al. [36] reported that there was no significant correlation between laboratory-rated user performance and accelerometer-rated actual prosthetic use. Therefore, any discussion about the use of prostheses in daily life should be carefully considered.

Cusack et al. reported that during AO, matching of the observation target and the (prosthetic) hand promotes planning-related activities in the brain and strategies for shoulder/elbow movements [12, 37]. Therefore, using highly immersive VR to observe the prosthetic user or the prosthetic simulator control may lead to increased promotion of motor learning.

Additionally, efficient prosthetic training may contribute to reducing the training period and medical costs. We believe that reducing the rejection of prostheses and increasing the use of prostheses in daily life will lead to an improvement in the quality of life of upper-limb amputees. Furthermore, AO using VR can be performed in the early stage of rehabilitation for upper-limb amputees when it is still difficult to perform prosthesis operations during stump formation and skin grafting.

### Study limitations

The subjects of this study were healthy young people. Therefore, our results may differ from studies involving upper-limb amputees. Future clinical studies should involve upper-limb amputees to confirm the effectiveness of the methods. Furthermore, in this study, the BBT and BKT were both used as the training and testing tasks. Therefore, it is necessary to carefully discuss whether this result can be generalized to other tasks. Which will be considered in future studies. Finally, we the BBT and BKT lasted for 10 min in each session; research on the optimal observation time is needed.

## Conclusions

In this study, we examined whether AO training using VR was effective in acquiring short-term BP prosthetic control skills. Bilateral manual dexterity in VR1 and VR3 groups showed enhanced prosthetic control compared to the control group. VR1 and VR3 additionally showed higher immersion scores during AO than Tablet3, suggesting that the higher the immersion score is, the shorter the execution time of the BKT task.

Therefore, our findings suggest that VR-based AO training may be effective in acquiring bilateral BP prosthetic control, which requires a more three-dimensional operation. This result may help improve the quality of daily life for upper-limb amputees.

## Data Availability

The datasets used and/or analysed during the current study are available from the corresponding author upon reasonable request.

## Abbreviations

BP prosthesis: Body-powered prosthesis
VR: Virtual reality
AO: Action observation
BBT: Box and Block Test
BKT: Bowknot task
VAS: Visual analogue scale
Tablet: Tablet device
